# Language Separation in Bidialectal Speakers: Evidence From Eye Tracking

**DOI:** 10.3389/fpsyg.2018.01394

**Published:** 2018-08-20

**Authors:** Björn Lundquist, Øystein A. Vangsnes

**Affiliations:** ^1^Department of Linguistics, UiT The Arctic University of Norway, Tromsø, Norway; ^2^Department of Language, Literature, Mathematics and Interpreting, Western Norway University of Applied Sciences, Sogndal, Norway

**Keywords:** eye tracking, Visual World Paradigm, Norwegian, bidialectalism, bilingualism, linguistic variation, morphological gender, language change

## Abstract

The aim of this study was to find out how people process the dialectal variation encountered in the daily linguistic input. We conducted an eye tracking study (Visual Word Paradigm) that targeted the online processing of grammatical gender markers. Three different groups of Norwegian speakers took part in the experiment: one group of students from the capital Oslo, and two groups of dialect speakers of the Sogn dialect of Western Norway. One Sogn group was defined as “stable dialect speakers,” and one as “unstable dialect speakers,” based on a background questionnaire. The students participated in two eye tracking experiments each, one conducted in the their own dialect, and one in the other dialect (i.e., Sogn dialect for the Oslo students, and Oslo dialect for the Sogn students). The gender systems in the two dialects differ: the Sogn dialect makes an obligatory three-gender split (Masculine, Feminine, and Neuter) whereas the Oslo dialect only obligatorily makes a two gender distinction. The research question was whether speakers could use gender markers to predict the upcoming target noun in both local and non-local dialect mode, and furthermore, if they correctly could adjust their expectations based on dialect mode. The results showed that the Sogn speakers could predict upcoming linguistic material both in the local and Oslo dialect, but only the stable group were able to adjust their predictions based on the dialect mode. The unstable group applied a more general Oslo-compatible parsing to both the local and the non-local dialect. The Oslo speakers on the other hand were able to use gender markers as predictors only in their own dialect. We argue that the stable Sogn group should be treated as a bilingual group, as they show native-like skills in both varieties, while the unstable Sogn group can be seen as accommodated monolinguals, in that they treat the two varieties as sharing an underspecified grammar. The Oslo group on the other hand lacks sufficient competence in the other dialect to make use of grammatical markers to make predictions.

## 1. Introduction

The linguistic input to the language learner contains noise and variation at all linguistic levels, and it is a challenge for the learner to sort out when the variation corresponds to differences in meaning, and when it simply reflects within-speaker or between-speaker variation. Some variation in the input is however more systematic than other, as some forms may be restricted to certain registers, dialects or in the most obvious case, certain languages. It is beyond doubt that a child growing up in a bilingual environment will learn that certain word orders, morphological classes, phonemic contrasts and lexical items are restricted to only one of the languages in the environment, and will be able to correctly build up two sets of grammars and lexicons (even though there is leakage and transfer between grammars and lexicons during acquisition and beyond). We have however very little knowledge about how and when a language learner figures out that the input should be sorted into different languages. How different does the input from two speakers have to be for a language learner (child or adult) to treat it as coming from two different “languages”? This question is specifically relevant in a linguistic environment where speakers are constantly exposed to different dialects with small differences in grammars (syntax, morphology, phonology) and lexicon.

In this article we focus on the Norwegian dialect situation, and we ask the question whether listeners access a different grammar when they listen to their own dialect compared to a dialect with a partially different morpho-syntactic feature inventory. We present the first (as far as we know) eye tracking study that simultaneously targets gender processing in the speakers' native and non-native dialect. The tested dialect pairing crucially differs minimally in the gender distinctions: one of the dialects, the Oslo dialect, has a two gender division (Neuter and Common Gender), and the other one, the Sogn dialect, has an obligatory three gender division (Neuter, Masculine, and Feminine). We test whether speakers are able to process gender cues equally well in both dialect modes, and further if they can adjust their language processing according to dialect mode. Several factors may of course influence the outcome of a test like this, and in this study, we target the factors *dialect stability* and *minority vs. majority dialect*. We test three groups of speakers: a group of “stable” speakers of a minority dialect, a group of “unstable” speakers of a minority dialect, and a group of speakers of the majority dialect. As we will see, both factors have an effect on the results. The speakers of the minority dialect have a rich input of the majority dialect from both written and spoken media (and mobility), while the group of majority dialect speakers have a much more limited input of the minority dialect. This makes it hard to distinguish the effect of sociolinguistic pressures (e.g., high vs. low status dialect) from the effect of pure amount of input. Although we will not make a strong point about this in the current article, the differences we find between the groups can be explained from amount of input only, and we have no need to invoke factors of language sociology into our analysis.

The motivations for carrying out this research are many. First, there is a growing number of studies on bilingual populations, both in linguistics and psychology, which target potential cognitive effects of bilingualism (Bialystok, 2001; Sebastián-Gallés et al., 2012; Abboub et al., 2015) and even anatomical differences between mono- and bilingual speakers' brains (Luk et al., 2011; Abutalebi and Green, 2016). As long as we do not have a clear idea of what counts as a bilingual experience, the results (and especially null-results) from these studies are hard to interpret: basically everyone can understand a closely related dialect or master different registers, which potentially leaves us with no control group to test the bilinguals against. This study is a first attempt to pin down what counts as a bilingual language experience.

Another reason for carrying out this study is to get a better understanding of the change in the grammatical gender system that is currently taking place in many Norwegian dialects (Lødrup, 2011; Rodina and Westergaard, 2015; Lundquist et al., 2016). The old three-gender system is being replaced with a two gender system, where the feminine and masculine gender has merged to a common gender, using the exponents from the masculine paradigm, as in the Oslo dialect. The explanation for this change may simply be the fact that dialect speakers are more exposed to the Oslo system than previously, either from spoken media (TV/internet), written media/books or increased mobility. However, the change could also be driven by language internal forces, such as simplification of the feature system. At the present stage, we know very little about how dialect speakers parse and analyse the Oslo dialect. Do they treat the input from the Oslo dialect as native language input, or as L2 input. If the input is treated as “foreign,” we expect that its influence on the local dialect should be relatively small, possible similar to the influence on Norwegian from English (which is also abundant in the input through internet, movies, computer games and music). However, if the input from other dialects is treated as the same “language” as the local one, change may be much faster. The current study directly targets the question whether speakers associate the distribution of the gender markers with individual dialects, or if they average over the total input of Norwegian. The results should help us get a better understanding of the general mechanisms behind *language change* and especially how sociolinguistic notions like dialect leveling and geographical diffusion (Trudgill, 1986; Kerswill, 2004) relate to the notion of L1 attrition, as frequently studied in the L2/bilingualism research (Schmid, 2011). Is the dialect leveling the result of unavoidable leakage between grammars in a bilingual/bi-lectal mind, or is it a result of an increasen inclusion of other dialects into the native register, thus altering the properties of the “L1” input? This research is an obvious meeting ground for sociolinguistic studies on variation, and more psycholinguistically oriented studies on L2 acquisition and L1 attrition, and the results should be highly relevant for both strands of research.

## 2. Background

### 2.1. Bilingualism, bidilalectalism, and language separation

Infants can already at birth separate a foreign language from their mother tongue, at least when the two languages are prosodically different (Mehler et al., 1996). At the age of 2 months, an infant can tell apart the mother tongue from a prosodically similar language. Even children growing up in bilingual homes will separate the two languages, again even in situations when the two languages are rhythmically similar, as most clearly has been shown by Bosch and Sebastián-Gallés (2001) in their studies of Spanish-Catalan (future bilingual) infants. However, the fact that infants can perceptually distinguish two languages in the input does not necessarily mean that they also build up separate grammars for the two languages. There has been a debate in the field of bilingualism from the late seventies about bilingual children's language separation. Volterra and Taeschner (1978) proposed that bilingual children first develop a joint system (Single System Hypothesis), and not until later differentiate the two languages. Later research has however shown that children already at the one-word stage clearly separates the two languages (Quay, 1995), and there is now a large body of evidence that bilingual children build up separate grammars for the two languages during early acquisition (De Houwer, 1995; Meisel, 2004; Snape and Kupisch, 2017, chap. 6). However, there is also ample evidence of cross-linguistic influence during bilingual language acquisition, i.e., the children may temporarily transfer structures from one of the languages into the other language (Genesee et al., 1995; Hulk and Müller, 2000). Although this may go on for a while, early bilinguals will eventually be native-like in both languages in the normal case. Transfer also takes place during later second language learning, and in this case, some L1 traits may be very persistent in the L2 (Odlin, 2013 and references therein). Late L2 learners often fail to develop fully native-like competence of the second language (Abrahamsson and Hyltenstam, 2009; Sorace and Filiaci, 2006), which can be seen in their production, but also their comprehension: psycholinguistic research has shown that L2 speakers are slower than first language speakers at language processing (see Kaan, 2014 for an overview, and discussion below).

As mentioned above, some studies of bilingualism have targeted speakers of two closely related languages, e.g., Spanish and Catalan. However, we have little knowledge about how different two varieties have to be for the language learner to treat them as two different languages. Although linguists have generally given up trying to draw a strict line between language and dialect (or sociolect), most researchers would intuitively agree that the relation between, for instance, British English and American English is somehow different than the relation between British English and Norwegian, the second pair obviously being different languages but not necessarily the British-American English pair. However, whether a speaker builds up different grammars for different varieties, be it sociolects, dialects or languages, must be an empirical question, which can only be answered with the help of standard psycholinguistic studies, and not by our intuitions about what constitutes a language.

Whereas psycholinguistic methods are frequently used in studies of bilingual speakers and L2 learners, only very few studies have applied psycholinguistic methods to investigate the linguistic competence in speakers who have command of more than one dialect. One recent example is Kirk et al. (2018), who showed that speakers of Scottish English performed similar to bilingual speakers in a switching task involving switching between the local dialect and standard English, indicating that at least on a lexical level, bidialectals show similar behavior as bilinguals. However, Melinger (2018) found no trace of language separation in a Scottish English population in a picture-word interference task. Both of these studies looked for signs of bilingualism in the lexicon. In contrast, we apply psycholinguistic methods to investigate morpho-syntactic processing in speakers who either have competence in or substantial exposure to two dialects.

### 2.2. The Norwegian dialect situation and the grammatical gender system

In the Norwegian language situation children typically grow up learning a local dialect, and later encounter other dialects or a regional/national standard in school, in written texts, or as a result of mobility. Most speakers will not only acquire a receptive second dialect competence, but will also be able to adjust their speech to approach a regional standard or perceived majority dialect if necessary, although the social norm is to not adjust (Vikør, 2001). Similar dialect situations are presumably found in most countries, but what makes Norway special is that the standardized written language comes in two varieties, Bokmål and Nynorsk, and the significance of this for our study will emerge below.

Norway is often divided into four major dialect areas; Eastern Norwegian, Western Norwegian, Central Norwegian (“Trøndersk”), and Northern Norwegian. These major dialect areas each contain several dialects, which may differ along several linguistic dimensions (see Mæhlum and Røyneland, 2012 for an accessible and up to date overview). This article will focus on the Oslo dialect, which is part of the Eastern Norwegian dialect area, and the Sogn dialect, which is part of the Western Norwegian dialect area.

The Western Norwegian dialect area is the main area in which Nynorsk is the default schooling language: about 90% of all children who have Nynorsk as their primary language in school live in this area (Source: public online database provided by The Norwegian Directory of Education). In turn, this means that Bokmål is the schooling language in most other parts of Norway, including the Oslo area. The relevance of this to the present study is that Nynorsk has an obligatory three-gender system, and although three genders is allowed in Bokmål too, this variety can also be used with only two genders.

Table 1 shows the gender markers in indefinite noun phrases in Nynorsk and Bokmål. We split Bokmål in two varieties: popular and conservative Bokmål (see also Vikør, 2015). What we call “popular” Bokmål embodies morphological exponents associated with rural and working class dialects of Eastern Norway (and is often referred to as “radical”), and we contrast this to “conservative” Bokmål which is a writing convention associated with more formal and high class speech and which lacks the three way gender distinction. Grammatical gender is visible on the article and the adjective in the indefinite noun phrase. However, the masculine-feminine distinction is not visible on adjectives in any written or spoken Norwegian varieties today (except for a couple of adjectives), whereas the masculine-neuter distinction is obligatory in all dialects. The three-way gender distinction is thus mainly visible on the indefinite article. The Feminine-Masculine distinction is also visible on some possessives, which will not be discussed here. Table 2 gives the gender forms in definite noun phrases. Again, the feminine-masculine distinction is less marked than the masucline-neuter distinction: both the definite article and the definite suffix carry special neuter marking in all three varieties, while only the definite suffix has a dedicated feminine exponent.

**Table 1:** Indefinite noun phrases in Feminine, Masculine and Neuter, in Nynorsk and popular and conservative Bokmål.

	**Masculine**	**Feminine**	**Neuter**
Nynorsk	ein raud hane	**ei** raud bok	**eit** raud-**t** hus
Popular bokmål	en rød hane	**ei** rød bok	**et** rød-**t** hus
Conservative bokmål	en rød hane	en rød bok	**et** rød-**t** hus
English	a red rooster	a red book	a red house

*Gender forms that are different from the masculine are boldfaced*.

**Table 2:** Definite noun phrases in Feminine, Masculine and Neuter, in Nynorsk and popular and conservative Bokmål.

	**Masculine**	**Feminine**	**Neuter**
Nynorsk	den raud-e han-en	den raud-e bok-**a**	**det** raud-e hus-**et**
Popular bokmål	den rød-e han-en	den rød-e bok-**a**	**det** rød-e hus-**et**
Conservative bokmål	den rød-e han-en	den rød-e bok-en	**det** rød-e hus-**et**
English	the red rooster	the red book	the red house

*Gender forms that are different from the masculine are boldfaced*.

As will be most relevant for this article, the tables show that Bokmål allows for masculine forms of articles to be used with feminine nouns, thereby obscuring the feminine-masculine distinction, while Nynorsk still makes an obligatory three-way gender distinction on articles. Most Norwegian dialects still have three grammatical genders, but in several of the big cities, the feminine gender is either entirely missing (as in the second biggest city, Bergen), or visible only on bound affixes (Oslo, see e.g., Lødrup, 2011). As shown in recent studies (Lødrup, 2011; Rodina and Westergaard, 2015), the masculine-feminine distinction is currently disappearing in several Norwegian cities (Oslo, Trondheim, Tromsø). The change is mainly seen in the indefinite article: the masculine article *en/ein* is replacing the feminine article *ei*. *En/ein* can thus be used with both feminine and masculine nouns. The feminine form of the definite suffix has however remained: *en bok – bok-a* (a_*masc*._ book – book-the_*fem*._) in most varieties. The new system that is arising in the bigger cities Oslo, Trondheim and Tromsø thus has the conservative Bokmål indefinite paradigm, and the popular Bokmål definite paradigm. However, there are tendencies both in Oslo (see below) and Trondheim (Busterud et al., in press) to also replace the feminine definite suffix with a general masculine/common gender suffix.

The status of the two written systems is discussed in Vangsnes et al. (2017). It is important to note that Bokmål is by far the dominant written language in Norway, and newspapers, books (including translations), and subtitles for films and TV are mainly in Bokmål. Furthermore, only 12.2% of the students in Norwegian primary and secondary schools have Nynorsk as their primary language, and even in Western Norway, the cities are Bokmål dominated. Children who grow up in Nynorsk areas are therefore heavily exposed to Bokmål from early on, and they encounter Bokmål basically as soon as they learn to read, as books and comic books are often only available in Bokmål. Children in Bokmål areas will on the other hand not have much exposure to Nynorsk until they are required to study it in school from the age of 14. As argued by Vangsnes et al. (2017), the children in the Nynorsk areas qualify as a bidialectally literate group, as opposed to the children in the Bokmål areas.

The two written varieties are clearly very similar, but there are both lexical and morphological differences, also beyond the gender system. There are also several spelling differences that typically mirror differences in pronunciation in spoken varieties of Norwegian: as a rule of thumb, in such cases Bokmål links up with pronunciation and grammar in Central Eastern Norway (the greater capital area) and other major cities whereas Nynorsk links up with the rural (and semi-rural) varieties in other parts of the country. However, due among other things to a substantial language planning effort to bring the two varieties closer to each other and eventually merge, both of the written varieties allow for some variation in the marking of inflectional categories. This now abandoned official language policy lasted throughout the twentieth century.

### 2.3. L1 and L2 processing of gender and speaker information

Psycholinguistic research has shown that we make use of pragmatic, lexical, morpho-syntactic and prosodic cues to rapidly interpret the incoming speech stream, and possibly to even predict upcoming linguistic material to speed up comprehension (see Huettig and Mani, 2016; Kuperberg and Jaeger, 2016 for recent overviews). However, psycholinguistic research has also shown that second language (L2) speakers are slower than first language speakers at processing, and possibly fail to use morphosyntactic and prosodic cues in online comprehension (see Kaan, 2014 and references therein). Grammatical gender is a linguistic feature that is highly suitable for the study of online comprehension; especially in studying how speakers make use of inflectional material to predict upcoming linguistic material. Although some recent studies have questioned if or to which extent speakers really engage in prediction during language processing (see Yan et al., 2017; Nieuwland et al., 2018), the current pool of evidence from L2 research still shows that L2 speakers are poorer at making use of morpho-syntactic features in their language processing. If the differences between L1 and L2 speakers should be stated as a qualitatively difference in predictive processing, or simply speed of lexical integration and online processing, is of no importance to our current research (again, see Kaan, 2014 for discussion). Relevant to our study are also studies on integration of speaker information into the interpretation of the message. In an ERP-study, Van Berkum et al. (2008) showed that listeners take into account gender and social class of the speakers when interpreting sentences, and this happens simultaneously with the interpretation of the message. Presumably, listeners should also take into account dialect variation in similar ways as e.g., variation tied to social class. It is however not obvious if the speaker effects should be equally prominent for morpho-syntactic features like grammatical gender and agreement as for the pragmatic and lexico-semantic features that Van Berkum et al. (2008) tested. Presumably, this should depend on whether listeners have strong association between a specific dialect and the use of specific morpho-syntactic features in that dialect. Still, it is not clear if semantic predictions based on cloze probabilities are qualitatively the same as predictions based on syntactic agreement relations, such as those between an article and a noun, and neither is it clear if L1-L2 difference can be found in the same extent for semantic and morpho-syntactic predictions. Intuitively, effects of surprisal triggered by semantic or pragmatic mismatches, should be found in L2 speakers as well as L1 speakers, but see Martin et al. (2013) for ERP results suggesting that semantic predictions are weaker in L2 speakers than L1 speakers.

One common paradigm for studying gender processing is the Visual World Paradigm (VWP) with eye tracking (see e.g., Dahan et al., 2000; Grüter et al., 2012; Dussias et al., 2013; Hopp, 2016; Lundquist et al., 2016). As has been shown in previous eye tracking VWP studies, L1 speakers are superior to L2 speakers in making use of gender marking in their language processing (Lew-Williams and Fernald, 2010). However, balanced simultaneous bilingual speakers may be able to make predictive use of gender markers in both languages, and adjust their predictions based on language exposed to (see Lemmerth and Hopp, 2018 for support). With the use of the Visual World Paradigm, we can thus test if dialect speakers behave like (simultaneous) bilinguals, in being able to take into account the gender inflection present on prenominal articles in their processing of the two varieties, or if they ignore dialect mode, and apply the native parsing strategy to both varieties (with equal success). Alternatively, they may simply fail to make predictions in the non-native dialect, thus behaving like early L2 learners. Yet another possibility is that the dialect speakers parse the native input with the grammar of the non-native dialect, something that may happen during dialect change due to large exposure to a national standard. The results from one previous Norwegian dialect study of gender processing (Lundquist et al., 2016) suggest that the latter is true, at least in dialect speakers that use Bokmål as their written standard. The dialect speakers in Lundquist et al. (2016) used the three-gender system consistently in their productions, but were not able to predict upcoming nouns preceded by a masculine or feminine article. These results suggested that the inconsistent use of the relevant articles encountered in written texts and in spoken input from other dialects had affected the processing of the native dialect. That is, even though the relevant articles in fact were reliable predictors in the native dialect, the participants did not expect a consistent use of the articles. Importantly though, the neuter article, which is used consistently in both written Bokmål and the dialect tested, was treated as a reliable predictor. However, the three-gender speakers were not able to use the masculine article as a predictor, even when it was contrasted with the neuter; a distinction that should be reliable. This again suggest that the inconsistency in the input has far-reaching effects. The result from this study also suggested that a small number of speakers who had entirely stopped using the feminine gender, had developed a stronger masculine-neuter distinction, so that the masculine article *was* a reliable predictor when contrasted with neuter, but not with feminine.

## 3. Research questions

The present study investigates how systematic dialect variation is perceived. Do speakers activate different grammars when they encounter dialects with partially different grammars, or do they parse all dialect input with one and the same underspecified grammar/parser? Furthermore, do dialect speakers have sufficient knowledge of the other dialect to make use of morphosyntactic markers in their online comprehension? We approach these questions by looking at the processing of grammatical gender in two groups of speakers from Sogndal in the Sogn county, and one group from the capital Oslo. As was laid out above, Norwegian dialect speakers will encounter plenty of variation with respect to grammatical gender markers: feminine nouns may be preceded by either a feminine or a masculine indefinite article. The variation is however semi-systematic, in the sense that speakers of certain dialects use the masculine article for all non-neuter nouns (Bergen and Oslo), while speakers of other dialects consistently use feminine articles with feminine nouns. In addition, the variation is systematic in the written language: in Nynorsk, which is the written standard in Sogn, masculine indefinite articles are only used with masculine nouns and never with feminine nouns. In Bokmål, which is the written standard in the Oslo area, feminine articles are only optionally used with feminine nouns, and in most contexts for most speakers, masculine articles are used with all feminine nouns. Still, we do not know if speakers associate the variation in article use with different spoken dialects.

All the participants in our study encounter the feminine article in their input, and the feminine article is then always followed by a feminine noun. All the partipants also encounter masculine articles followed by feminine nouns. The frequency in the input of the feminine article however differ massively for the Sogn participants and the Oslo participants. The Sogn participants encounter feminine nouns with masculine articles in written Bokmål, in spoken media (TV, radio) and when they interact with speakers of other dialects. This is still a fairly large part of their input. The Oslo participants will more seldom encounter the feminine article, and this either in written Nynorsk, but also occasionally in Bokmål, or from speakers of other dialects. Still, this is a non-negligible part of their input. If we assume that speakers do not link the article use to different dialects, all the participants should in principle behave alike in a language perception task: they should all be able to predict that only feminine nouns can follow a feminine article, and that either a feminine or a masculine noun can follow a masculine article, irrespective of dialect. However, if they do ascribe different grammatical properties to different dialects, they should adjust their prediction based on the current dialect input, just as a bilingual speaker can adjust expectations on upcoming linguistic material based on the language in the speech situation.

Based on the background of previous gender processing studies conducted on monolingual speakers, bilinguals and L2 learners, we can now investigate if the dialect speakers show similar processing profiles to any of those groups. Depending on the linguistic background, a dialect speaker could in principle show any of the following four processing profiles:

1. The true bilingual: A speaker who can correctly adjust their predictions based on input language/dialect.
2. The true/ignorant monolingual: A speaker who uses morphosyntactic features in their online comprehension in their L1, but fails to do so in the other language/dialect.
3. The monolingual generalizer: A speaker who imposes the gender system of the L1 onto the other language/dialect.
4. The accommodated monolingual: A speaker who imposes the gender system of the other dialect onto the L1.

Whether a dialect speaker ends up matching any of the profiles above should depend on several factors. First, if a dialect speaker has had little exposure to the other dialects s/he should have small chances of making use of the grammatical feature in that dialect, and then show a behavior incompatible with either profile 1 or 4 above. Furthermore, for this speaker to be able to apply the L1 feature distinctions to the other dialect (profile 3), the other dialect better be fairly similar to the L1, otherwise phonological and lexical differences may trigger general processing difficulties. We expect to find the bilingual profile in speakers who have a fairly balanced input of the two dialects, but who also associate the different dialects with different contexts and groups of speakers. Speakers who on the other hand have grown up in an environment where the dialects are less clearly separated should be more likely to apply the same parsing strategy to all inputs, ending up as monolingual generalizers or accommodated monolinguals.

The most important evidence will come from the contrast between the feminine and masculine article. The core question is whether speakers still expect that only masculine nouns (and not feminine nouns) can follow a masculine article. We predict that the speakers of a strict three-gender system should expect only masculine nouns to follow the masculine article, at least when they listen to the local dialect. If they behave like true bilinguals, they should however adjust their expectations when they listen to input from a potential two gender dialect, and have equal expectations of a masculine or a feminine noun to follow the masculine article. Speakers of a two gender dialect, should in principle also be able to adjust their expectations accordingly when they listen to a three-gender dialect, at least if the speakers have sufficient knowledge of the three gender dialect.

## 4. Participants

Seventy-seven Norwegian high school students (17–18 years) participated in the study. Thirty-four of them were students from a high school in the Oslo area (Lambertseter vgs) who all identified themselves as speakers of the Oslo dialect. Oslo was chosen as a target location since the children growing up there speak a dialect where the masculine-feminine distinction is almost fully absent today. Forty-three of the participant were students from the Sogndal vgs (high school) in Western Norway. Sogndal was chosen because the dialect spoken there (Sogn dialect) still has a clear three way gender distinction. As the students in Sogndal are schooled in Nynorsk, they also have the three-gender system reinforced by reading and writing. The participants from Sogndal all identified themselves as speakers of Western Norwegian, but for several reasons, they differed in their relation to the local Sogn dialect: some of the participants came from neighboring towns where the dialects differed slightly from the dialect in Sogndal (but still would count as “Sogn” dialect), some had one or two parents that spoke other dialects. To isolate a core group of stable Sogn dialect speakers, we split the students into two groups: one group that considered themselves to speak both like the other students of the school and their parents, and one group of speakers that indicated that they either did not speak like the other students of the school, or their parents. The first group we label the Stable Sogn group (*n* = 21), and the other group the Unstable Sogn group (*n* = 22). We chose to define the groups based on these criteria rather than on more standard criteria like place of birth/childhood, and origin of parents, since it is hard to geographically delimit the dialect isoglosses, and also to assess the perceived proximity (or identity) between dialects. Furthermore, the dialect background of the parents may not necessarily mirror how they speak to their children. Given previous reports on the ongoing language change, it was important to isolate a group of young Norwegian speakers that were likely to have maintained the old three-gender system, which we judged to be speakers in a Nynorsk area from stable dialect background. This is the group we call the Stable group.

In addition to the eye tracking experiment, the participants filled in a background questionnaire targeting language background and language attitudes, and participated in a production test, targeting the relevant gender forms (see next section). The background questionnaire consisted of 10 statements which the participants were asked to evaluate from 1 (completely false) to 10 (fully true). The Oslo students reported to have little problem reading Nynorsk (the statement “I find it hard to understand written Nynorsk” received a mean score a of 3.69, i.e., close to completely false), but overall they found writing in Nynorsk hard, or at least harder than writing in English (the statement “I find it harder to write in Nynorsk than English” received a mean score of 7.93). These students thus seem to have no big difficulties understanding Nynorsk, which is unsurprising given the extreme typological proximity, but producing it in writing is harder, presumably due to different spelling systems and a partly different set of morphological rules. Both groups of Sogndal students preferred reading in Nynorsk to Bokmål, but the preference was significantly stronger in the Stable group. From the background questionnaire, it is also worth mentioning that the Oslo students reported that they spoke more like their peers than their parents, while the Sogn groups assessed themselves as speaking in a way that was equally similar to their parents as their peers.

### 4.1. Background production test

All the participants took part in a production test after the eye tracking study. This test was solely included to make sure that our participants actually matched the expected production profile as described in available dialect grammars (e.g., Mæhlum and Røyneland, 2012). This was of special relevance since, as mentioned in section 2.2, the gender system is currently changing in many dialects, and previous studies have shown that the change has proceeded surprisingly rapidly in the Bokmål areas in and around Trondheim and Tromsø (Busterud et al., in press; Rodina and Westergaard, 2015). The production test was administered by a speaker of the local dialect to encourage the speakers to speak the local dialect. The test was set up as a picture naming task, where two pictures first appeared on the screen accompanied with a spoken question “What do you see?”. After that, one of the pictures disappeared, and the question “What disappeared?” was asked (see Rodina and Westergaard, 2015 for a similar task). The first question triggered responses in the indefinite form (e.g, “I see a book and a bird”), and the second question triggered answers in the definite form (e.g., “The book disappeared”). Some item pairs differed in color (e.g., a black book and a yellow book). We thus received information about both the use of the form of the indefinite article and the definite suffix. The test included 11 indefinite feminine targets, and seven definite feminine targets. Neuter and masculine nouns were included as well, and the participants performed fully target-like on these items.

**Figure 1:**
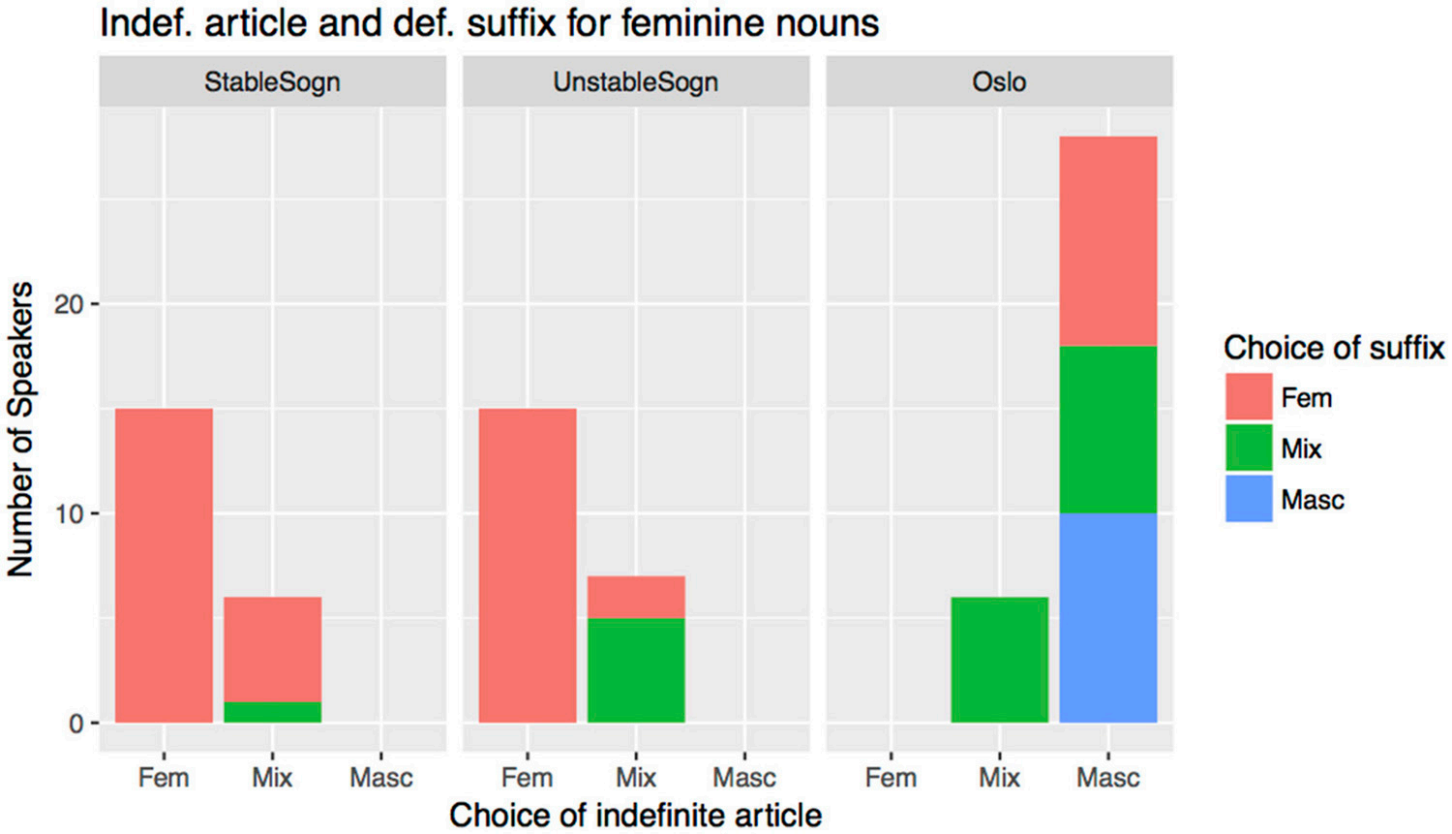
Production results, stable Sogn (*n* = 21), unstable Sogn (*n* = 22) and Oslo (*n* = 34), 11 indef. fem nouns, 7 def. fem nouns. The choice of gender of the indefinite article is shown on the x-axis, and the choice of gender on the definite suffix is color coded. The diagram shows the production results for each individual, and not the total number of feminine/masculine forms.

The results are shown in Figure 1. Around two thirds of the participants in the Sogn groups produced target like indefinite and definite feminine forms (15 of 21 and 15 of 22 participants for the stable and unstable group, respectively). A smaller group of Sogn speakers (1/3) mixed between masculine and feminine indefinite articles for the feminine nouns. The mixers within the stable group were almost target like in the production of the definite suffix, while the mixers in the unstable group also mixed feminine and masculine definite endings. Overall, < 10% of the target indefinite feminine articles *ei* were realized as masculine articles (*ein*).

The Oslo production results look diametrically opposite: the large majority (27 out of 33) consistently use the masculine indefinite article with feminine nouns. Ten participants also consistently used the masculine definite suffix, and equally many consistently used the feminine definite suffix. The remaining participants mixed between masculine and feminine suffixes. Only six of the Oslo participants produced some feminine indefinite articles, but no one used the feminine indefinite article consistently with the feminine nouns. In total, <5% of the feminine nouns were preceded by a feminine article in the Oslo group.

The Oslo and the Sogn groups thus differ massively in their production patterns. The Sogn groups were close to target-like in their production, suggesting that the Sogn dialect has not been affected by the change to the same extent as the Tromsø and Trondheim dialects. Furthermore, the total amount of feminine markers in the Oslo group was surprisingly small, specially the definite markers, suggesting that the feminine nouns no longer exists as a noun group for most young Oslo speakers. The two Sogn groups differ only minimally (in definite suffixes). However, it's worth pointing out that not even the stable Sogn group is completely target like in their use of feminine articles, and also that feminine exponents are still produced by the young Oslo speakers. We will return to the errors in the Sogn group is the discussion of language change in section 10.

## 5. Eye tracking experiment: material and design

Two versions of a Visual World experiment were used in the test: one with spoken stimuli from the Sogn dialect, and one with stimuli from the Oslo dialect. The two experiments were identical modulo the spoken stimuli, and consisted of in total 64 images selected from Cycowicz et al. (1997), arranged in 128 items over four lists (32 items per list). Each item consisted of two images, and the two images either depicted objects with different grammatical gender (diff. condition) or the same grammatical gender (same condition). We had four different gender conditions: Neuter (target) vs. Masculine (distractor), Masculine vs. Neuter, Feminine vs. Masculine and Masculine vs. Feminine. See Table 3 for example of same and different condition. The counterbalancing was done in the following way: we created 16 sets of images (4 images in each): 8 sets targeting the Neuter-Mascunline distinction and 8 sets targeting the Masculine - Feminine distinction. The four objects in each set matched in animacy, frequency and scale, i.e., the corresponding real world objects are of roughly the same size (e.g., “Book” and “Plate” rather than “Book” and “House”). For each set we created 8 items, so that every image appeared as the target in both the same and different conditions, and distractor in both same and different conditions. The 8 items were distributed over the four lists, two items in each list, with each image/noun appearing only once per list. The participants did the test twice, once in local mode and once in non-local mode, and they were assigned different lists in each mode. The list assigned in the second round, never contained the same item (or picture pairing) as in the first list, e.g., from the set consisting of *bear* (Masc.), *pig* (Masc.), *rhino* (Neut.) and *donkey* (Neut.), the items *bear (Targ.) - pig (Dist.)* and *rhino* (Targ.) - *donkey* (Dist.) appeared in one mode, and the items *bear* (Targ.) - *rhino* (Dist.) and *donkey* (Targ.) - *pig* (Dist.) appeared in the other, making it impossible to predict target noun based on items in the first round. All the nouns in the experiment are cognates in the two dialects, and they do not differ in gender in the standardized written languages (i.e., all the feminine nouns in the test have the definite feminine ending *-a* in both Nynorsk and Popular Bokmål). Even though the feminine gender on the indefinite article is rarely used in the Oslo dialect, all the feminine nouns were recorded with the feminine article. The two dialects modes thus only differed with respect to the dialect pronunciation, and not with respect to overt gender cues in the sound stimuli.

**Table 3:** The four gender conditions, in the different and same condition. Examples below presented in their Bokmål forms.

	**Diff. condition**		**Same condition**	

	**Targ**.	**Dist**.	**Targ**.	**Dist**.
Neuter-Masc	et hus	en bil	et hus	et tog
(Targ. - Dist.)	*a house* (N)	*a car* (M)	*a house* (N)	*a train* (N)
Masc-Neuter	en bil	et tog	en bil	en sykkel
(Targ. - Dist.)	*a car* (M)	*a train* (N)	*a bil* (M)	*a bike* (M)
Fem-Masc	ei bok	en vase	ei bok	ei flaske
(Targ. - Dist.)	*a book* (F)	*a vase* (M)	*a book* (F)	*a bottle* (M)
Masc-Fem	en trompet	ei bok	en trompet	en vase
(Targ. - Dist.)	*a trumpet* (M)	*a book* (F)	*a trumpet* (M)	*a vase* (M)

## 6. Recap and predictions

The design of the study is admittedly quite complex, since it involves three groups of speakers, each doing a test with four gender contrasts in two different dialects modes. This work is in many ways exploratory, since there is little research on how dialect variation is processed, or even how gender is processed in Norwegian. However, based on the results in Lundquist et al. (2016), we can make some predictions about how the groups of speakers in the current experiment will behave. As mentioned in section 2.3, the three-gender speakers in the Bokmål area Troms failed to use both feminine and masculine in their online comprehension, even when masculine was contrasted with a neuter distractor. There were however some indications that speakers with a stable two gender system could make use of the masculine-neuter contrast. The result from Lundquist et al. (2016) is summarized in Table 4 arranged after the gender contrasts used in the current study, with results from both the three-gender speakers (a large group of participants) and the two-gender speakers (a smaller group of speakers who were native speakers of other dialects).

**Table 4:** Results from Lundquist et al. (2016). Checkmark indicates that the participants could use the relevant gender marker in the relevant context to predict the target noun, and the asterisk indicates that they could not.

	**Neuter-Masc**	**Masc-Neuter**	**Fem-Masc**	**Masc-Fem**
3 gender (Bokmål)	✓	^*^	^*^	^*^
2 gender (Bokmål)	✓	✓(?)	^*^	^*^

*The first part of the column name is the gender of the target image, and the second part is the name of the competitor. The colors likewise indicate the result for simple comparison with the predictions in Table 5 and results in Table 10. Green, observed/likely prediction; Yellow, weaker/less likely prediction; Red, no prediction*.

The current experiment differ slightly in the design from the one in Lundquist et al. (2016): the previous had four objects in each stimulus (one target, three distractors), while the current one has two (target and distractor). It is in principle possible that the effects of gender predictions are easier to see in a two-picture design. Further, Lundquist et al. (2016) did not manipulate the dialect mode, but used only stimulus in the local Troms dialect mode. Based on the previous results, we expect that all groups in our current study will be able to make predictive use of the neuter article, at least in the local mode. We also find it more likely that the Sogn groups should be sensitive to the Fem-Masc and even Masc-Fem contrasts in the local mode, given the reliable input of all three articles in both the spoken dialect and the written Nynorsk. If anything, the Unstable group could show less reliable patterns, possibly more similar to the Troms participants: just like the Troms participants, they use the feminine gender in a target-like way, but they are also used to feminine nouns with masculine articles in their input. Our hypothesis is that the stable group should be less susceptible to the Bokmål/Oslo input, and therefore be more likely to make predictive use of the masculine article, even when contrasted with the feminine. The Oslo group should behave like the two-gender group in Lundquist et al. (2016). We lay out the predicted patterns for the local dialect modes in Table 5, where we mark how likely the groups are to make use of the gender markers as predictive cues in the respective gender conditions.

**Table 5:** Predicted results for the three groups in the local mode, stated in how likely speakers are to make use of the gender feature to locate the target.

	**Neuter-Masc**	**Masc-Neuter**	**Fem-Masc**	**Masc-Fem**
Sogn Stable	Very likely	Likely	Likely	Likely
Sogn Unstable	Very likely	Likely	Likely	Less likely
Oslo	Very likely	Likely	Unlikely	Unlikely

*The first part of the column name is the gender of the target image, and the second part is the name of the competitor. The colors indicate predicted results, for simple comparison with the previous results in Table 4 and the results in Table 10*.

The patterns for the non-local mode is harder to predict. If the three groups all behave like “naive” monolinguals, and treat the non-local dialect as foreign language, then they should not be able to make use of any of the gender markers in their comprehension, i.e., the corresponding non-local mode table would be all red. If they on the other hand treat is as their own L1, i.e., if they are monolingual generalizers, the result for the local and non-local mode should be identical for the three groups. We hypothesize that the three groups should differ in their sensitivity to the dialect manipulation. The stable group is most likely to treat the Oslo dialect as qualitatively different from their own dialect: they have grown up with the local dialect and the Nynorsk writing system, but have subsequently encountered the Oslo dialect, often in connection with the Bokmål writing system, with its frequent absence of feminine markers. They should therefore be more likely to expect both feminine and masculine nouns after the masculine article in the Oslo mode. The unstable group is less likely to make a categorical dialect distinction given their more mixed background. The Oslo group are unlikely to pick up the masculine-feminine distinction in the Sogn dialect, since they have relatively little input of this dialect. The small amount of input the Sogn dialect may also lead to overall processing difficulties for the Oslo group.

## 7. Procedure and data collection

The procedure of the eye tracking experiment was the following: Each trial starts with a preview of the two pictures which lasts for 2000 ms. The preview is accompanied by a voice naming the two objects, in the relevant dialect. The objects were named by a pre-recorded voice without any articles or other elements revealing the gender of the referent (e.g., “bear” and “donkey”). After this, the two objects disappear, and a cartoon character turns up in the middle of the screen, saying “I'm hiding behind a [noun].” At the onset of the article, the two pictures reappear. We use a gaze contingent paradigm, and when the target is fixated, the cartoon character turns up in place of the target (800 ms after fixation). The length of the article is always 500 ms, i.e., in every item there is exactly 500 ms from the onset of the article to the onset of the noun. The voice of the cartoon character and the voice that named the referents were not the same, but they were of the same dialect, and they were recorded by female speakers. We chose the set-up with the cartoon character rather than a more standard set-up (“Look at the X”) for several reasons: first, the gender distinction between masculine and feminine is not seen on the pre-nominal definite article, but only the indefinite article. Using a “Look at…” paradigm would be infelicitous with indefinite articles; the spoken stimulus *Look at a bear* with two known objects on the screen would be pragmatically marked. Furthermore, the cartoon character functions as a reliable fixation point, making sure that the participants gaze is fixated right in the middle of the screen at the onset of the article. Finally, the cartoon character minimally changed poses and facial expression throughout the experiment, which helped the participants stay focused and entertained throughout an otherwise highly repetitive task. The eye tracking experiment was conducted with an SMI RED 500 eye-tracker, at a sampling rate of 250 Hz.

The data collection took place in two high schools, Sogndal VGS in Sogndal, and Lambertseter VGS in Oslo. Arrangements were made with teachers and administrators at the schools in advance so that we would have access to students and two rooms for running tests. Each experimental session started with the participant signing a consent form and filling in a sheet of background information. This was followed by the eye tracking test in one of the dialect modes (counterbalanced), which lasted for 5–6 min, including calibration. The participants were told that they were going to do the test twice, in two different dialect at the very beginning. Otherwise, a minimum of instructions were given to the participants (e.g., “Just look at the object that the cartoon figure is hiding behind”). After the first round, the participants did a filler task (prosodic perception), which lasted for about 7 min, which was followed by the second eye tracking round in the other dialect mode. In the second round, the items were from a different list, i.e., with different picture combinations for the items, as was described in section 5. After the second round, the participants took part in a short production study (described in section 4.1). The whole test lasted for about 30 min. The participants received 50 NOK (7 USD) each, which was given to a shared school/class account.

## 8. Analysis

To detect if participants make use of the gender feature of the indefinite article in their online processing, we looked at the participants' fixations of the target object in contexts where there is only one referent of the target gender on the screen (test/different condition) compared to contexts where there are two objects of the target gender on screen (same/control condition). That is, we compared the fixations in a condition where the gender information on the indefinite article can help the participant predict the upcoming target noun (different condition) with a condition where it cannot (same condition). We investigated the effect of the dialect mode for the different groups, as well as the effect of the gender contrast (Table 3).

**Figure 2:**
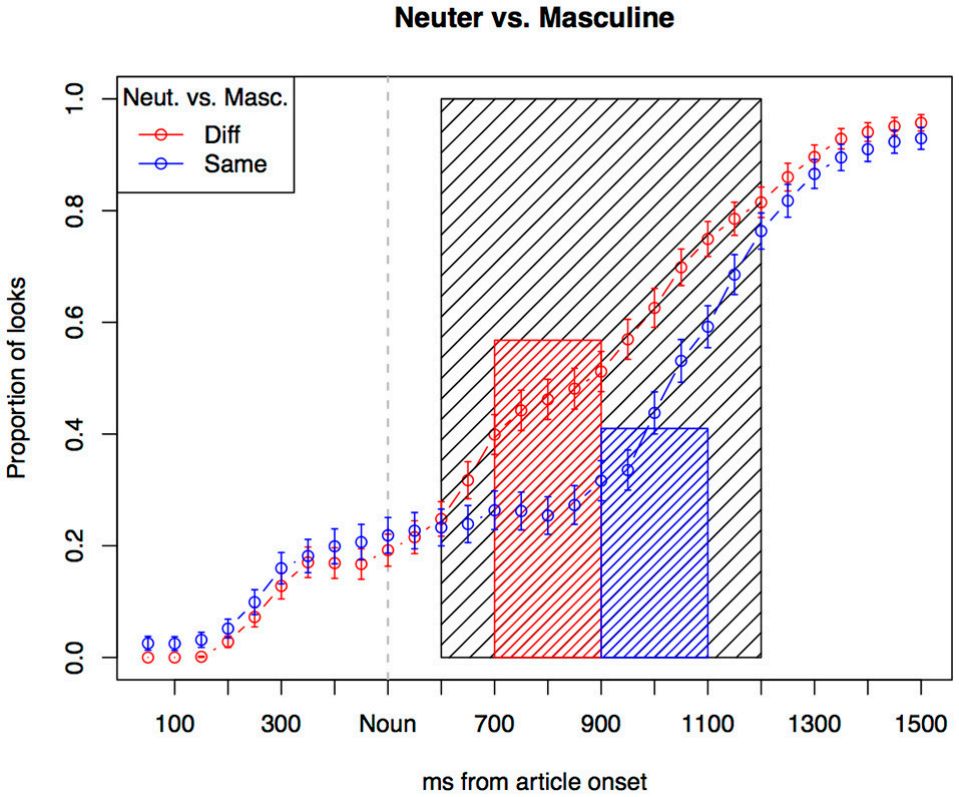
Illustration of the relevant time window for analysis. The gray box marks the window span for the analysis. We average the proportion of looks to the target (looks to target in relation to total registered looks to either target, distractor or white space) in a time window from 600 to 1,200 ms. The lines show the proportion of looks to target for each 50 ms time slot, and the bars show the average of looks within the whole time window, which is the dependent variable in the subsequent analyses.

In the analysis, we focus on a time span starting 600 ms after the article onset and ending 1,200 ms after article onset. We chose this fairly late and large time region for several reasons: the three articles all share the same onset, and are not reliably distinguished until 300 ms after onset. Furthermore, we expect the participants to take 200–300 ms to carry out a saccade. We thus do not expect any looks triggered by the gender marker to take place before 500–600 ms after the article onset. Fixations after 1,200 ms are presumably triggered solely by the target word. See Figure 2 for an illustration of the relation between the dependent variable and the temporal unfolding of the experimental items. Furthermore, the articles differ in their acoustic properties across the dialects, with time of differentiation possibly taking place at different times. By making the time window fairly large, we are able to compare the gender effect across dialects and gender markers.

In analysing the results, we start with a model targeting overall group differences with respect to dialect mode (Mode, 2 levels: Sogn, Oslo) and same/different condition (Cond., two levels: Same, Different). We analyse the data using logistic mixed effects regression (with the lmer package in R, Bates et al., 2015). The dependent variable is the number of fixations at the target relative to the number of fixations at the distractor and white space. After establishing group differences, we analyse each group separately. In cases where we find effects of mode within the group, we analyse each mode separately, in order to pin down the potential effect of condition and gender contrast (Gender, 4 levels, see Table 3) in both modes. All models include the maximally complex converging random effects structures, which minimally included random intercepts for Participant and Item, by-Participant slopes for Cond and Gender and by-Item slopes for Cond (following guidelines in Barr et al., 2013). The coefficients from the models for the three groups, split into two modes in cases where necessary, are provided in Tables 6–**9** including standard errors, *z*-values and *p*-values.

**Table 6:** Model (glmer, logistic) for the variables Cond, Group and Mode.

**Fixed Effect**	**β**	***SE***	***z***	***p***
(Intercept)	0.3041	0.1913	1.589	0.11204
CondSame	−0.6243	0.1258	−4.963	6.96*e*−07^***^
GroupOslo	−0.6728	0.2358	−2.853	0.00433^**^
GroupUnstable	−0.4073	0.2574	−1.583	0.11353
ModeOslo	−0.3055	0.1422	−2.149	0.03166^*^
CondSame: GroupOslo	0.5336	0.1293	4.128	3.67*e*−05^***^
Condsame: GroupUnstable	0.1742	0.1413	1.233	0.21763
Condsame: ModeOslo	0.1415	0.1655	0.855	0.39256
GroupOslo: ModeOslo	0.4445	0.1576	2.821	0.00479^**^
GroupUnstable: ModeOslo	0.2926	0.1717	1.703	0.08848
CondSame: GroupOslo: ModeOslo	−0.2551	0.1738	−1.467	0.14229
CondSame: GroupUnstable: ModeOslo	−0.0915	0.1898	−0.482	0.62979

*Model coefficients for the proportion of looks to target in relation to looks at distractor and white space, 600–1,200 ms after article onset. Number of obs: 4,850, Participants: 76, Items: 32, Conditions: 2, Modes: 2. Intercept is the different condition, Stable group in Sogn mode. The model includes random intercepts for Participant and Item, and by-Participant and by-Item slopes for Cond and Mode, as well as Cond x Mode interactions.Significance levels*:

*** *p < 0.001*;

** *p < 0.01*;

* *p < 0.05*.

In short, we modeled the data at three different levels: (1) Group and Mode, targeting effects of Same/Different gender manipulations (Cond), but ignoring the specific gender contrasts, (2) Mode effects within the different groups, and (3) specific effects of gender within the individual groups and modes (in case we found an effect of Mode within the group). Graphs and regression tables are included at the first and third levels, and effects of Mode for the individual groups (level 2) are reported *p* and χ^2^ values from likelihood ratio tests. In the Group analysis (level 1), the stable Sogn group is the reference group/intercept, and in the individual Group/Mode analyses (level 3), the Neuter-Masculine gender condition is the intercept.

## 9. Results

### 9.1. Overall group and mode differences

**Figure 3:**
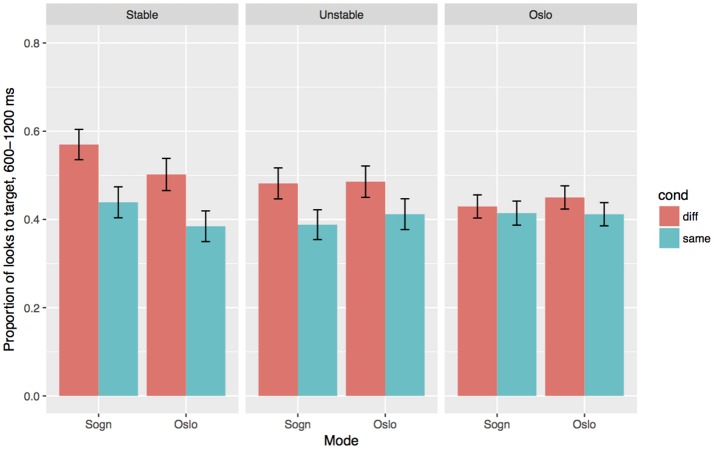
Effects of Condition (Different/test vs. Same/control) and Mode for the three groups. Error bars indicate 95% confidence intervals.

The overall differences between the three groups in the two dialect modes are shown in Figure 3, and the coefficients and *p*-values are given in Table 6. We find significant main effects of Cond, Group and Mode, as well as interactions between Cond and Group, and Cond and Mode. The two Sogn groups show an effect of Cond in the expected direction independent of dialect mode (β = 0.62, *SE* = 0.12, *p* < 0.001), i.e., they fixate more on the target when the article can be used for predicting the target. The Stable group also overall have more fixations at the target in the Sogn mode compared to the Oslo mode (β = 0.3, *SE* = 0.14, *p* < 0.05), while the effect of Mode is smaller or absent in the Unstable group (as shown in the interaction between Mode and Group: β = 0.29, *SE* = 0.17, *p* = 0.088). The Oslo group differ from the two Sogn groups in several respects: the Oslo group overall have less target fixations in the Sogn mode compared to the stable Sogn group (β = 0.67, *SE* = 0.24, *p* < 0.01), and the difference between Same and Different conditions is smaller or completely absent (as shown in the interaction between Cond and Group:Oslo: (β = 0.53, *SE* = 0.13, *p* < 0.001). In addition, we find an interaction between Mode and Group:Oslo (β = 0.44, *SE* = 0.16, *p* < 0.01), which suggests that the Oslo group, unsurprisingly, does not look less to the target in the Oslo mode, in comparison to the Sogn mode; if anything, they look more to the target in their local mode. In short, we see that the two Sogn groups can make use of the gender information to locate the target in both dialects modes, although the Stable group overall have more looks to target in the relevant time frame in the Sogn mode. The Oslo group shows no clear signs of making predictive use of the gender marker in the Sogn mode, and only a small effect is seen in the Oslo mode, which we will look more carefully at below. Below we go through the results for the three groups separately and add the factor Gender in the analyses, in order to estimate the effect of the individual gender markers in the different groups and modes.

### 9.2. The stable sogn group

**Figure 4:**
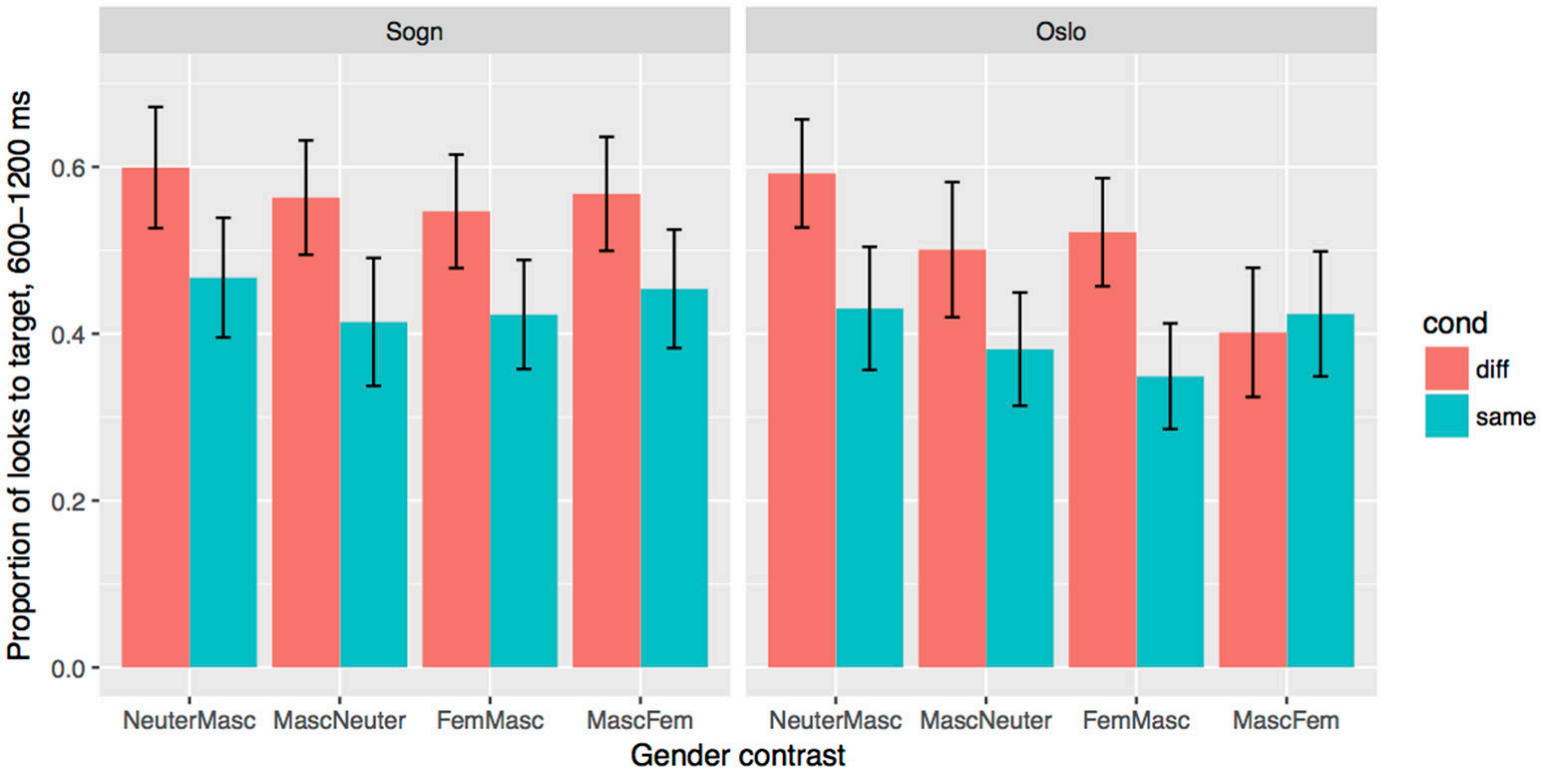
Effects of Condition (Different/test vs. Same/control) and Gender in the two modes for the stable Sogn group. Error bars indicate 95% confidence intervals.

The mode effects in the Stable group reported above come out as an interaction between Mode and Gender χ^2^ = 19, *df* = 4, *p* < 0.001), as well as three-way interaction between Mode, Cond and Gender χ^2^ = 29, *df* = 8, *p* < 0.001). We will look at the results for two modes separately, to assess how the gender contrasts affect processing in the two modes. The results for the two modes are given in Figure 4, and the coefficients from the models are given in Table 7. In the Sogn mode, we find a main effect of Cond (β = −0.77, *SE* = 0.24, *p* < 0.01), but no effect of Gender, i.e., the gender cue on the article is used equally in all four gender conditions. The results are different in the Oslo mode. Here we still find a strong effect of Cond (β = −0.-77, *SE* = 0.26, *p* < 0.01), but also effects of Gender: MascFem: β = −1.06, *SE* = 0.22, *p* < 0.001, MascNeuter: β = −0.58, *SE* = 0.11, *p* < 0.001, FemMasc: β = −0.43, *SE* = 0.22, *p* = 0.051. There is furthermore an interaction between Cond and GenderContrast MascFem: β = −0.94, *SE* = 0.38, *p* < 0.01. *Post-hoc* reveal that there indeed is no effect of the Same/Different manipulation in the MascFem gender contrast. That is, the gender marker was used predictively in all condition except the MascFem condition, and furthermore, there were more fixations at the target when the target gender was neuter, especially compared to the masculine targets.

**Table 7:** Stable group.

**Fixed Effect**	**Sogn Mode**				**Oslo Mode**			

	β	***SE***	***z***	***p***	β	**SE**	***z***	***p***
(Intercept)	0.543	0.269	2.020	0.04341^*^	0.510	0.233	2.191	0.02847^*^
CondSame	−0.774	0.236	−3.280	0.00104^**^	−0.770	0.261	−2.945	0.00323^**^
TargetGenderFemMasc	−0.363	0.264	−1.376	0.16890	−0.432	0.222	−1.946	0.05171.
TargetGenderMascFem	−0.209	0.267	−0.784	0.43318	−1.063	0.225	−4.728	2.26*e*−06^***^
TargetGenderMascNeuter	−0.302	0.233	−1.295	0.19515	−0.580	0.112	−5.191	2.10*e*−07^***^
CondSame: TargetGenderFemMasc	0.261	0.299	0.871	0.38351	−0.008	0.337	−0.025	0.98001
CondSame: TargetGenderMascFem	0.162	0.300	0.541	0.58821	0.939	0.338	2.779	0.00546^**^
CondSame: TargetGenderMascNeuter	0.090	0.209	0.434	0.66431	0.241	0.261	0.924	0.35572

*(glmer, logistic) for the effects Cond and TargetGender. Model coefficients for the proportion of looks to target in relation to looks at distractor and white space, 600–1,200 ms after article onset. Number of obs: 704 (Sogn) and 672 (Oslo), Participants: 21 (Sogn), 20 (Oslo), Items: 32, Conditions: 2, Modes: 2. Intercept is the different NeuterMasc. The model includes random intercepts for Participant and Item, and by-Participant for Cond and TargetGender and by-Item slopes for Cond. Significance levels*:

*** *p < 0.001*;

** *p < 0.01*;

* *p < 0.05*.

### 9.3. The unstable sogn group

**Figure 5:**
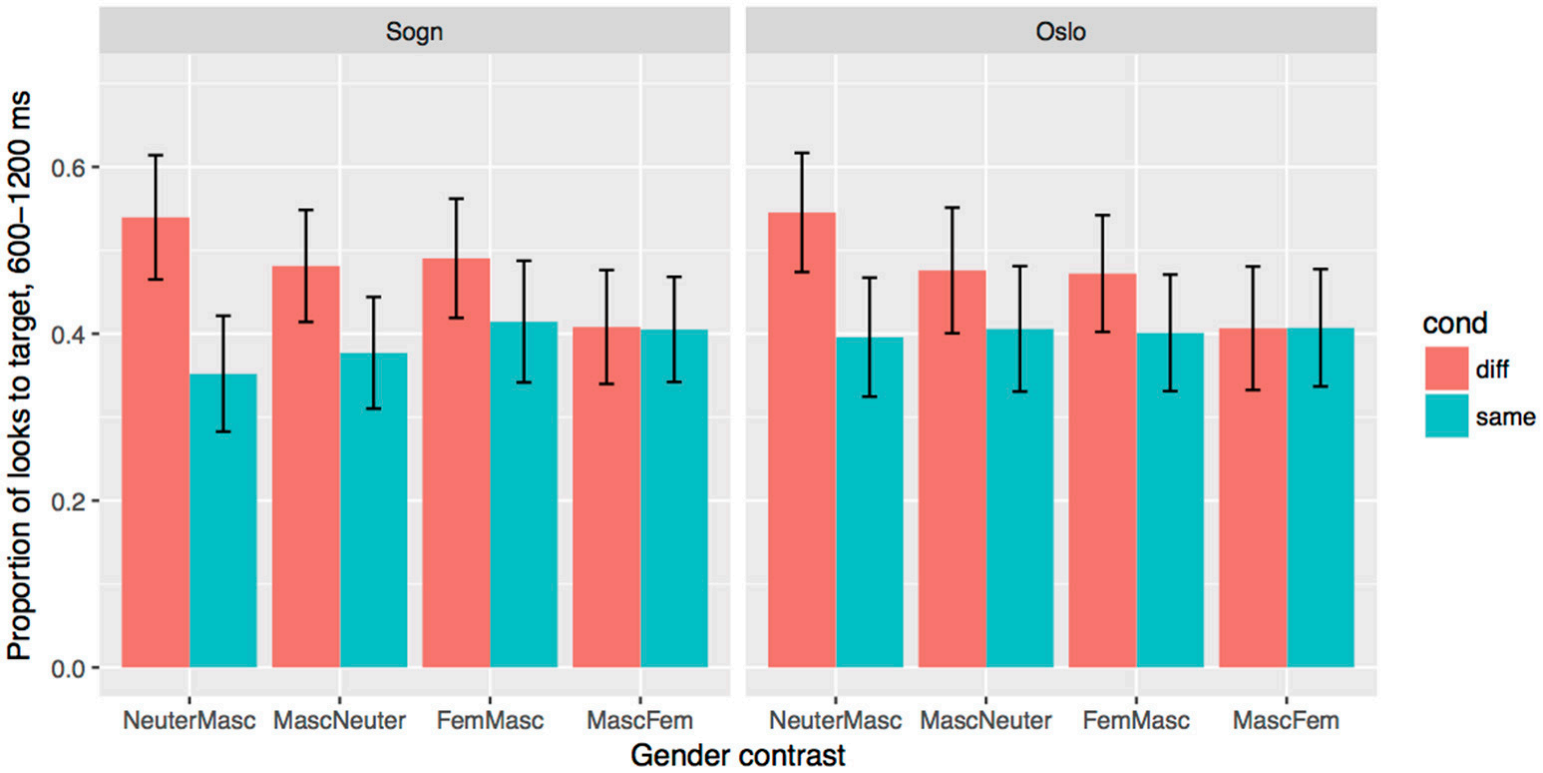
Effects of Condition (Different/test vs. Same/control) and Gender in the two modes for the Unstable Sogn group. Error bars indicate 95% confidence intervals.

In contrast to the Stable group, the Unstable group shows no main effect of Mode, nor an interaction between Mode and Condition or Mode and Gender. We illustrate the results in the two different modes in Figure 5, and report the coefficients and *p*-values from a model including both modes (Table 8). We still find a strong effect of Cond (β = −0.85, *SE* = 0.23, *p* < 0.001), but also an effect of Gender MascFem (β = −0.61, *SE* = 0.27, *p* < 0.05), as well as an interaction between MascFem and Cond (β = −0.75, *SE* = 0.33, *p* < 0.05). In fact, we see no effect of Cond in the MascFem gender condition, just as in the Oslo Mode in the Stable group. The Unstable group thus seem to be able to make predictions based on gender in both modes in all gender contrasts except MascFem. In other words, they find feminine and masculine nouns equally plausible as complements of the masculine article, independent of dialect mode.

**Table 8:** Unstable group.

**Fixed Effect**	**β**	***SE***	***z***	***z***
(Intercept)	0.2256	0.3168	0.712	0.476333
CondSame	−0.8497	0.2325	−3.654	0.000258^***^
TargetGenderFemMasc	−0.3952	0.2419	−1.634	0.102309
TargetGenderMascFem	−0.6141	0.2677	−2.294	0.021810^*^
TargetGenderMascNeuter	−0.2661	0.1632	−1.631	0.102896
CondSame: TargetGenderFemMasc	0.4781	0.3460	1.382	0.167085
CondSame: TargetGender MascFem	0.7498	0.3329	2.252	0.024298^*^
CondSame: TargetGenderMasc Neuter	0.2808	0.2780	1.010	0.312404

*Model (glmer, logistic) for the effects Cond and TargetGender. Model coefficients for the proportion of looks to target in relation to looks at distractor and white space, 600–1,200 ms after article onset. Number of obs: 1,408, Participants: 22, Items: 32, Conditions:2, Modes: 2. Intercept is the different condition, NeuterMasc gender. The model includes random intercepts for Participant and Item, and by-Participant slopes for Cond and TargetGender (including interactions) and by-Item slopes for Cond. Significance levels*:

*** *p < 0.001*;

^**^p < 0.01;

* *p < 0.05*.

### 9.4. The Oslo group

**Figure 6:**
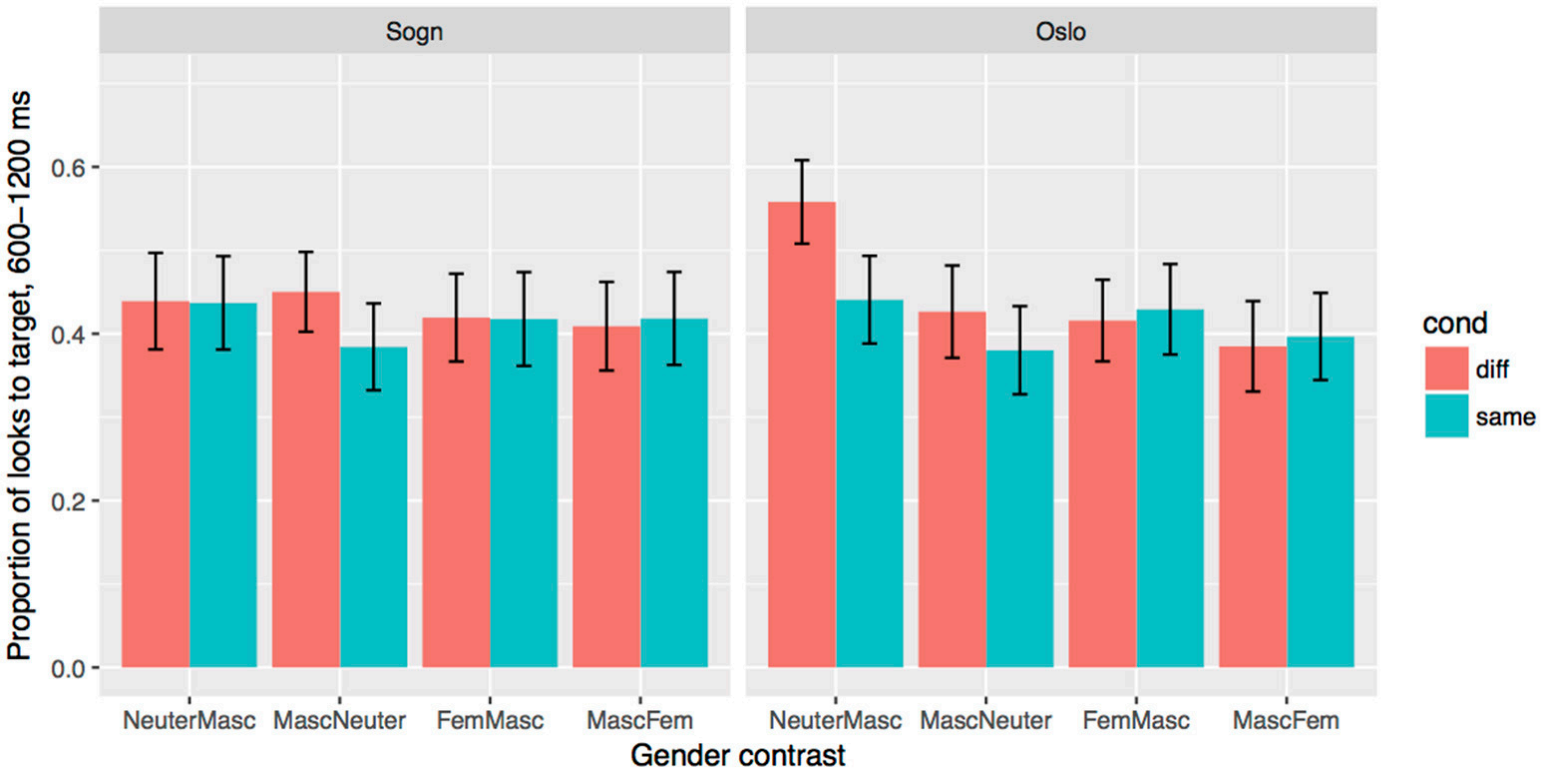
Effects of Condition (Different/test vs. Same/control) and Gender in the two modes for the Oslo group. Error bars indicate 95% confidence intervals.

In the Oslo group, we find an effect of Mode, which just as in the Stable group comes out as an interaction between Mode and Gender χ^2^ = 20.6, *df* = 4, *p* < 0.001), and a three-way interaction between Mode, Gender and Cond (χ^2^ = 20.5, *df* = 8, *p* < 0.001). In the Sogn mode, we see no effect of Cond. That is, the participants seem not to be able to make use of the gender information on the article to predict the target noun. As Figure 6 shows, there seem to be a small effect of Cond in the MascNeuter gender contrast, but this is not significant (see coefficients in Table 9). In the Oslo mode, we see a clear effect of Cond (β = −0.67, *SE* = 0.19, *p* < 0.001), but also effects of TargetGender (*p* < 0.001 for all Gender contrasts), as well as Cond by TargetGender interactions for all GenderContrasts (all *p* values smaller than 0.05). *Post-hoc* tests reveal that only the NeuterMasc contrast has an effect of Cond; that is, only articles with neuter gender marking can be used predictively.

**Table 9:** The Oslo group.

**Fixed Effect**	**Sogn Mode**				**Oslo Mode**			

	β	**SE**	***z***	***p***	β	**SE**	**t**	***p***
(Intercept)	−0.428	0.2486	−1.722	0.0851.	0.333	0.186	1.787	0.073965.
condsame	0.034	0.2698	0.127	0.8988	−0.671	0.189	−3.537	0.000405^***^
TargetGenderFemMasc	−0.090	0.2396	−0.377	0.7065	−0.768	0.212	−3.624	0.000290^***^
TargetGenderMascFem	−0.096	0.2466	−0.388	0.6979	−0.942	0.237	−3.971	7.16*e*−05^***^
TargetGenderMascNeuter	0.181	0.1956	0.926	0.3547	−0.712	0.153	−4.642	3.46*e*−06^***^
condsame: TargetGenderFemMasc	−0.004	0.3540	−0.012	0.9905	0.729	0.292	2.493	0.012659^*^
condsame: TargetGenderMascFem	0.063	0.3594	0.177	0.8595	0.716	0.298	2.402	0.016290^*^
condsame: TargetGenderMascNeuter	−0.505241	0.330290	−1.530	0.1261	0.487	0.208	2.340	0.019275^*^

*Model coefficients for the proportion of looks to target in relation to looks at distractor and white space, 600-1200 ms after article onset. Number of obs: 1056 (Sogn), 1074 (Oslo), Participants: 33, Items: 32, Conditions:2, Modes: 2. Intercept is the different condition, NeuterMasc gender. The model includes random intercepts for Participant and Item, and by-Participant slopes for Cond and TargetGender (including interactions between Cond and TargetGender) and by-Item slopes for Cond. Significance levels*:

*** *p < 0.001*;

^**^p < 0.01;

* *p < 0.05*.

## 10. General discussion

The results from the three groups show that only two of them, the Stable Sogn group and the Oslo group, show effects of dialect mode, but they show it in different ways. The Unstable Sogn group behaved identically in the two modes. We will discuss the causes of the mode effects first, and afterwards look at the absence of mode effects in the Unstable group. We summarize the results in Table 10:

**Table 10:** Summary of the result, stated in terms of the strength of the different-same manipulation, i.e., the effect on looks to target as conditioned by seeing only one gender matching gender object on the screen.

	**Neuter-Masc**	**Masc-Neuter**	**Fem-Masc**	**Masc-Fem**
Stable: Local	Strong effect	Strong effect	Strong effect	Strong effect
Stable: Non-local	Strong effect	Strong effect	Strong effect	No effect
Unstable: Local	Strong effect	Weaker effect	Weaker effect	No effect
Unstable: Non-local	Strong effect	Weaker effect	Weaker effect	No effect
Oslo: Local	Strong effect	No effect?	No effect	No effect
Oslo: Non-local	No effect	No effect	No effect	No effect

*The colors code the strength of the effect, for simple comparison with Tables 4 and 5*.

The results show that the stable Sogn group behaves like a bilingual group, i.e., they show what we called the processing profile 1 in section 3. They make use of gender markers to make predictions in both their first dialect (Sogn) and their second dialect (Bokmål, Oslo). We see however two different effects of dialect mode. First, they are overall faster to locate the target in their first dialect compared to their second dialect. This is what we would expect from a group of unbalanced bilinguals. The second mode effect is more interesting: the participants correctly adjust their reliance on the gender cues according to dialect mode. In the local/Sogn mode, they treat the three gender markers as equally reliable predictors: a neuter noun is expected only after a neuter article, a feminine noun is expected only after a feminine article, and a masculine noun is expected only after a masculine article. In the Oslo mode, they correctly adjust their expectations: after a masculine article, a feminine or masculine noun is equally expected. They are thus aware that the masculine indefinite article is not a reliable cue for masculine nouns in Bokmål/Oslo dialect. This is evidence that this group treats the gender variation in the input as conditioned by dialect: the use of masculine articles with feminine nouns is restricted to a certain dialect register, leaving the first/local dialect unaffected, at least when it comes to online comprehension. The results from the stable group can easily be explained from the participants language background. They have grown up surrounded by a stable and fairly homogenous three gender dialect, and they have been schooled in Nynorsk, which makes an obligatory three gender distinction. They have also been aware, at least since they started reading, that there is another written variety, Bokmål, which they encounter in abundance through books, comic books, and subtitled TV. This written variety is further closely associated with the spoken linguistic input from TV and radio. It should be fairly easy for these speakers to develop an awareness about which linguistic features belong to which variety/dialect.

The mode effects in the Oslo group are of a different kind, and they match profile 2 (“the true monolingual”). This group can still make use of the available gender contrasts in their native dialect (i.e., Neuter-Masc), although they show no signs of awareness of the Masc-Fem distinction. The effect for the Masc-Neuter gender contrast was however significantly smaller then the Neuter-Masc condition, and possibly completely absent. That is, the neuter article triggered looks to the neuter noun, while the masculine article did not obviously trigger looks to the masculine noun, even when the distractor noun was obviously non-masculine (i.e., neuter). Similar effects were found in Lundquist et al. (2016) (and also in other ongoing studies at the University of Oslo and the University of Tromsø). One possible explanation for this is the following: the previous three-gender system was fairly balanced, although masculine is the most frequent, at least when looking at type frequencies. When the feminine and masculine gender merges into one gender category, around 80% of the nouns end up in the masculine/common gender group. This seems to lead to a reanalysis of the masculine gender as an unmarked/default gender, possibly void of gender features. In this situation, language users may simply stop making predictions based on the masculine article, since its domain is so large.

In the Sogn mode, the Oslo group is not able to make any predictions based on gender markers. They do not even transfer their knowledge of the Masc-Neuter distinction to facilitate comprehension of the Sogn dialect. Presumably, the young Oslo students only rarely encounter the Sogn dialect (or other Western Norwegian rural dialects), and although they are able to understand the dialect (due to linguistic proximity), they ignore the cues on the function words, and allocate their cognitive resources to the interpretation of the content words. This strategy works out well for them, as they are overall not noticeably slower in locating the target in the Sogn mode compared to the Oslo mode. The Sogn mode results can be compared to the results from L2 Spanish learners in Lew-Williams and Fernald (2010): grammatical cues are ignored, but the lexical knowledge is in place.

The Unstable group shows no effect of mode, neither as a main effect nor as an interaction with gender. They do however show a strong effect of condition, which indicates that they make use of the gender markers in their online comprehension, but they do so to the same extent in local and foreign dialect mode. This indicates that they parse the input in the same way and with the same efficiency in both modes. The interesting finding here is that they do not parse the input in the same way as the stable group parses the Sogn input, but rather as the stable group parses the Oslo input, i.e., they treat masculine and feminine nouns as equally plausible complements of a masculine article. In other words, they behave as what we call “accommodated monolinguals.” They thus parse the local input in an Oslo-compatible way, and not the other way around. Our interpretation of the results is the following: they treat the two variates/languages as one and the same, and they are thus unaware that feminine nouns with masculine articles only are licit in one of the varieties. They will solely note that feminine articles are optional in their language. However, they are still aware that the feminine article *ei* can only appear with a subset of the nouns (i.e., the feminine nouns). This is not surprising given that the feminine article never appears together with masculine or neuter nouns, independent of mode.

The findings reported on are clearly in need of an explanation, especially in terms of our notion dialect stability. Why does the factor that we loosely have called “stability” influence the way you sort the linguistic input into different “languages”? Or, stated differently, why does the group of stable speakers treat Oslo/Bokmål and Sogn/Nynorsk as two separate systems, while the unstable group does not? We will suggest that speakers may set the “language separation threshold” at different heights. Speakers who encounter a certain amount of variation in their primary linguistic input, for example, a child growing up with parents speaking different but closely related dialects, may treat the parents dialects as two possible outputs of one and the same underlying language, and may treat the points of variation just like other types of inter-individual variation, such as pitch, speech rate, or frequency of specific words and idioms. This child may set the “language separation threshold” higher than a child whose linguistic input contains basically no variation at all. By being exposed to variation early on, a speaker may set the language threshold high, and subsequently be more likely to incorporate features from other dialects into the first language, rather than building up a new grammar for subsequently encountered dialects. The speakers in our stable group are speakers who assess themselves as speaking in the same way as both their parents and their peers, i.e., a group that come from a linguistically uniform background. The speakers in the unstable group, are presumably part of the same speech community as the stable speakers, and their linguistic production is not hugely different from that of the stable group, but their background is different in one way or another. Either one or both of their parents are speakers of another Norwegian dialect, or they come from a town or village just outside of the core Sogn region, where the linguistic variety may be slightly different from the variety they hear from the majority of their school peers. In both situations, the speaker will have been constantly exposed to variation, but variation that may have been treated as within the limits of normal linguistic variation within the speech community.

What is important about the Norwegian language situation, is that speakers rarely modify or change their dialects according to speech situations. Most speakers are therefore exposed to massive linguistic variation, either in conversations in everyday life, or through mass media. There is little doubt that the exposure to variation ultimately will lead to dialect change or leveling, where features from the varieties that are most frequent in the input will spread. However, our hypothesis predicts that the change will be slower and less pervasive in dialects where speaker early on set the language threshold very low, i.e., where speakers early on conceive of their own variety as qualitatively different from other varieties. Just as an early bilingual or an L2 learner can keep the two languages apart, a “stable” dialect speaker should be able to avoid influence from other dialects or even a national standard, as long as the first/native variety is treated as a separate language/variety.

It is thus interesting to look at the change and variation we find in the Norwegian gender system against the results from the eye tracking results. As was stated in section 2.2, the gender systems in the Norwegian dialects is changing rapidly at the moment, and the results from our current production study show small signs of change both in the Stable and the Unstable group. We expect variation in the unstable group: following our hypothesis about language thresholds, we have to say that these speakers are constantly exposed to variation *within* their language, and this variation will lead to a more unstable production (here, both masculine and feminine articles with feminine nouns). However, we do not expect a similar degree of variation in the production of the Stable group, as their is less variation within their perceived local language boundaries. Even though the variation we found in the production experiment is relatively small in the Stable group, it is in need of an explanation. We can think of at least three sources for the variation. (1) If we think of the stable speakers as bilinguals, the variation may have its source in L2 influence on L1, as some researchers may claim is basically unavoidable (see Schmid and Köpke, 2007). (2) We could also think of the variation as a result of influence from a high prestige variant on local dialects, but this is not supported by recent sociolinguistic studies (see especially Sandøy et al., 2014). Finally (3), we could also think of the variation as a sign of a slow, internally caused change. These factors are not easy to tease apart, and all of them presumably play a role. The production data presented in section 4.1 however gives some support to the last explanation. The Masculine-Feminine distinction has slowly been eroding over the last 300–400 years, and Masc-Fem distinctions on adjectives have been gone for a long time in most dialects (in contrast to Neuter-Masc distinctions, which are still present on adjectives). When looking more closely at the production results, it turns out that all the errors involve cases where the article and the noun is separated by an uninflected adjective, e.g., *en gul bok* [“a(Masc) yellow book(Fem)”]. This could hint at problems with a “long-distance” gender agreement, rather than problems with the gender system itself. The speakers in the Unstable group on the other hand make errors when articles and nouns are adjacent (*en bok*) as well as non-adjacent, indicating that they accept and produce Masc-Fem pairs more freely.

The language separation is presumably strengthened by the properties of the two writing systems. As laid out in the background section, Bokmål, which is highly associated with the Oslo dialect, optionally (and rarely) uses the feminine article, while Nynorsk obligatory makes a three gender distinction on the articles. It is here worth comparing the findings from the current study with the findings from the gender studies from the Northern county Troms, where Bokmål is the standard written language (Lundquist et al., 2016). The participants in the Troms study showed gender prediction pattern that was almost identical to the Oslo group in the current study, even though they were often target like in their production of feminine articles. The exposure to feminine nouns with masculine articles in written material in their first written language may have caused these speakers to expect both masculine and feminine after masculine articles, even in spoken language from the local dialect. As mentioned in the background, the gender change seems to have progressed much further in the Northern dialects and in Trondheim, where Bokmål is the written standard. For future research it will make sense to look for dialect mode effects in dialect pairings that are both tied to the Bokmål written system, in order to estimate the effect of the written language.

It is worth pointing out that none of groups matched the profile we call “the monolingual generalizer,” i.e., a group that make use of the gender contrasts of the native dialect when parsing the foreign dialect. The reason why the Oslo group doesn't do this is presumably due to the fact that they still need to make quite an effort to understand the content words when listening to the Sogn dialect. They thus need to allocate more cognitive resources for interpreting the lexical material, leaving less resources for the functional material, including gender features. For the Stable Sogn group, the explanation may be more interesting, as they clearly have no problems parsing the Oslo stimuli. Their results show how sensitive the parser is to deviations in the input: as soon as speakers start to encounter masculine articles followed by feminine nouns, the speakers stop treating it as a reliable predictor. The results from the previous studies in Troms (Lundquist et al., 2016) suggest that is the this is the case in contexts where the local gender system is relatively reliable: only small amount of noise in the input is sufficient for treating grammatical markers as unreliable. In this sense the morpho-syntactic effects may qualitatively differ from semantic effects/pragmatic effects, where predictions about upcoming nouns have been shown even when cloze probabilities are well below 1.

Summarizing the findings from the three groups, we find that two of them are sensitive to the dialect mode in their language processing, but in different ways. The Oslo group fail to use gender marked articles as predictors for upcoming nouns when listening to the Sogn dialect. This is presumably due to the small amount of exposure the Sogn dialect in their daily input. The Stable Sogn group presumably have had enough input of the Oslo dialect to (a) be able to use the gender information on the articles to predict upcoming linguistic material, but also to (b) notice that the masculine gender is not a reliable predictor in masculine - feminine contrasts. Most importantly, we see that the stable group have different expectations on the Sogn and the Oslo input: they trust the Sogn three-gender system to be reliable, but not the Oslo system. Further, the group we call the unstable group, i.e., speakers that have a more varied linguistic background, fails to ascribe uncertainty to a certain dialect, and thus allows the inconsistency encountered in the non-local input affect their parsing of their local dialect as well. Overall, our findings show that predictions made on morpho-syntactic expectations is highly dependent on a strong regularity. It also suggest that speakers with little variation in their early input, are more likely ot build up strict grammars, with little room for optionality. A small amount of variation may however easily blur further fine-tuned distinctions between input from closely related varieties. We hypothesize that this may speed up language change, i.e., by being exposed to variation, language learners may fail to associate linguistic regularities within individual dialects/registers. Rather than calculating the probabilities of co-occurrence betweens articles and nouns in distinct dialects in the input, speakers average over the total amount of input, which lead the learners to treat varying gender markers as unpredictable signals. We hypothesize that this may speed up language change relative to instances of clear bi-lingual input, where separate grammars for the two languages in the input is more likely to develop.

## Ethics statement

This study was carried out in accordance with the recommendations of NSD (Norwegian Center for Research Data). No personal details were stored connected to names or other personal identifiers. All subjects gave written informed consent in accordance with the Declaration of Helsinki.

## Author contributions

BL and ØV: research formulation and data collection; BL: design of experiment, data analysis drafting of the article; ØV: contributions to revisions of the article.

### Conflict of interest statement

The authors declare that the research was conducted in the absence of any commercial or financial relationships that could be construed as a potential conflict of interest.

## References

[B1] Abboub N. Bijeljac-Babic R. Serres J. Nazzi T. (2015). On the importance of being bilingual: word stress processing in a context of segmental variability. J. Exp. Child Psychol. 132, 111–120. 10.1016/j.jecp.2014.12.004 25644083

[B2] Abrahamsson N. Hyltenstam K. (2009). Age of onset and nativelikeness in a second language: listener perception versus linguistic scrutiny. Lang. Learn. 59, 249–306. 10.1111/j.1467-9922.2009.00507.x

[B3] Abutalebi J. Green D. (2016). Neuroimaging of language control in bilinguals: neural adaptation and reserve. Bilingual. Lang. Cogn. 19, 689–698. 10.1017/S1366728916000225

[B4] Barr D. J. Levy R. Scheepers C. Tily H. J. (2013). Random effects structure for confirmatory hypothesis testing: keep it maximal. J. Mem. Lang. 68, 255–278. 10.1016/j.jml.2012.11.001 24403724 PMC3881361

[B5] Bates D. Mächler M. Bolker B. Walker S. (2015). Fitting linear mixed-effects models using lme4. J. Stat. Softw. 67, 1–48. 10.18637/jss.v067.i01

[B6] Bialystok E. (2001). Bilingualism in Development: Language, Literacy, and Cognition. Cambridge: Cambridge University Press.

[B7] Bosch L. Sebastián-Gallés N. (2001). Evidence of early language discrimination abilities in infants from bilingual environments. Infancy 2, 29–49. 10.1207/S15327078IN0201_3 33451225

[B8] Busterud G. Lohndal T. Rodina Y. Westergaard M. (in press). The loss of feminine gender in Norwegian: a dialect comparison. J. Compar. German. Linguist. 1–24.

[B9] Cycowicz Y. M. Friedman D. Rothstein M. Snodgrass J. G. (1997). Picture naming by young children: Norms for name agreement, familiarity, and visual complexity. J. Exp. Child Psychol. 65, 171–237. 10.1006/jecp.1996.2356 9169209

[B10] Dahan D. Swingley D. Tanenhaus M. K. Magnuson J. S. (2000). Linguistic gender and spoken-word recognition in French. J. Mem. Lang. 42, 465–480. 10.1006/jmla.1999.2688

[B11] De Houwer A. (1995). Bilingual language acquisition, in The Handbook of Child Language, eds Fletcher P. MacWhinney B. (Blackwell Publishing Ltd.), 219–250.

[B12] Dussias P. E. Valdés Kroff J. R. Guzzardo Tamargo R. E. Gerfen C. (2013). When gender and looking go hand in hand: grammatical gender processing in l2 spanish. Stud. Second Lang. Acquis. 35, 353–387. 10.1017/S0272263112000915

[B13] Genesee F. Nicoladis E. Paradis J. (1995). Language differentiation in early bilingual development. J. Child Lang. 22, 611–631. 10.1017/S0305000900009971 8789516

[B14] Grüter T. Lew-Williams C. Fernald A. (2012). Grammatical gender in l2: a production or a real-time processing problem? Second Lang. Res. 28, 191–215. 10.1177/0267658312437990 30319164 PMC6181447

[B15] Hopp H. (2016). Learning (not) to predict: grammatical gender processing in second language acquisition. Second Lang. Res. 32, 277–307. 10.1177/0267658315624960

[B16] Huettig F. Mani N. (2016). Is prediction necessary to understand language? probably not. Lang. Cogn. Neurosci. 31, 19–31. 10.1080/23273798.2015.1072223

[B17] Hulk A. Müller N. (2000). Bilingual first language acquisition at the interface between syntax and pragmatics. Bilingual. Lang. Cogn. 3, 227–244. 10.1017/S1366728900000353

[B18] Kaan E. (2014). Predictive sentence processing in l2 and l1: what is different? Linguis. Approaches Bilingual. 4, 257–282. 10.1075/lab.4.2.05kaa

[B19] Kerswill P. (2004). Chapter 26: Koineization and accommodation, in The Handbook of Language Variation and Change, eds Chambers J. K. Trudgill P. Schilling-Estes N. (Oxford: Blackwell Publishing Ltd.), 669–702.

[B20] Kirk N. W. Kempe V. Scott-Brown K. C. Philipp A. Declerck M. (2018). Can monolinguals be like bilinguals? evidence from dialect switching. Cognition 170, 164–178. 10.1016/j.cognition.2017.10.001 29024916

[B21] Kuperberg G. R. Jaeger T. F. (2016). What do we mean by prediction in language comprehension? Lang. Cogn. Neurosci. 31, 32–59. 10.1080/23273798.2015.1102299 27135040 PMC4850025

[B22] Lemmerth N. Hopp H. (2018). Gender processing in simultaneous and successive bilingual children: cross-linguistic lexical and syntactic influences. Lang. Acquis. 10.1080/10489223.2017.1391815. [Epub ahead of print].

[B23] Lew-Williams C. Fernald A. (2010). Real-time processing of gender-marked articles by native and non-native spanish speakers. J. Mem. Lang. 63, 447–464. 10.1016/j.jml.2010.07.003 21076648 PMC2976062

[B24] Lødrup H. (2011). Hvor mange genus er det i Oslo-dialekten? [how many genders are there in the Oslo dialect?]. Maal Minne 2, 120–136. Available online at: http://ojs.novus.no/index.php/MOM/article/view/330

[B25] Luk G. Bialystok E. Craik F. I. M. Grady C. L. (2011). Lifelong bilingualism maintains white matter integrity in older adults. J. Neurosci. 31, 16808–16813. 10.1523/JNEUROSCI.4563-11.2011 22090506 PMC3259110

[B26] Lundquist B. Rodina Y. Sekerina I. A. Westergaard M. (2016). Gender change in Norwegian dialects: comprehension is affected before production. Linguis. Vanguard 2, 1–15. 10.1515/lingvan-2016-0026

[B27] Mæhlum B. Røyneland U. (2012). Det Norske Dialektlandskapet. Oslo: Cappellen Damm.

[B28] Martin C. D. Thierry G. Kuipers J.-R. Boutonnet B. Foucart A. Costa A. (2013). Bilinguals reading in their second language do not predict upcoming words as native readers do. J. Mem. Lang. 69, 574–588. 10.1016/j.jml.2013.08.001

[B29] Mehler J. Dupoux E. Nazzi T. Dehaene-Lambertz G. (1996). Chapter 7: Coping with linguistic diversity: the infant's viewpoint, in Signal to Syntax: Bootstrapping From Speech to Grammar in Early Acquisition, eds Morgan J. L. Demuth K. (Cambridge, MA: The MIT Press), 101–116.

[B30] Meisel J. M. (2004). Chapter 3: The bilingual child, in The Handbook of Bilingualism, eds Bhatia T. K. Ritchie W. C. (Oxford: Blackwell), 91–112.

[B31] Melinger A. (2018). Distinguishing languages from dialects: a litmus test using the picture-word interference task. Cognition 172, 73–88. 10.1016/j.cognition.2017.12.006 29232596

[B32] Nieuwland M. Politzer-Ahles S. Heyselaar E. Segaert K. Darley E. Kazanina N.. (2018). Large-scale replication study reveals a limit on probabilistic prediction in language comprehension. eLife 7:e33468. 10.7554/eLife.33468 29631695 PMC5896878

[B33] Odlin T. (2013). Crosslinguistic influence in second language acquisition, in The Encyclopedia of Applied Linguistics, ed Chapelle C. A. (Oxford: Blackwell), 1–6.

[B34] Quay S. (1995). The bilingual lexicon: implications for studies of language choice. J. Child Lang. 22, 369–387. 10.1017/S0305000900009831 8550728

[B35] Rodina Y. Westergaard M. (2015). Grammatical gender in Norwegian: language acquisition and language change. J. Ger. Linguist. 27, 145–187. 10.1017/S1470542714000245

[B36] Sandøy H. Anderson R. L. Doublet M.-R. (2014). Chapter 11: The Bergen dialect splits in two, in Stability and Divergence in Language Contact. Factors and Mechanisms, eds Braunmüller K. en Höder S. Kühl K. (Amsterdam: John Benjamins Publishing Company), 239–264.

[B37] Schmid M. (2011). Language Attrition. Cambridge: Cambridge University Press.

[B38] Schmid M. S. Köpke B. (2007). Chapter 1: Bilingualism and attrition, in Language Attrition: Theoretical Perspectives, eds Köpke B. Schmid M. S. Keijzer M. Dostert S. (Amsterdam; Philadelphia, PA: John Benjamins), 1–7.

[B39] Sebastián-Gallés N. Albareda-Castellot B. Weikum W. M. Werker J. F. (2012). A bilingual advantage in visual language discrimination in infancy. Psychol. Sci. 23, 994–999. 10.1177/0956797612436817 22810164

[B40] Snape N. Kupisch T. (2017). Second Language Acquisition. London: Palgrave.

[B41] Sorace A. Filiaci F. (2006). Anaphora resolution in near-native speakers of Italian. Second Lang. Res. 22, 339–368. 10.1191/0267658306sr271oa

[B42] Trudgill P. (1986). Dialects in Contact. Oxford: Blackwell.

[B43] Van Berkum J. Van den Brink D. Tesink C. M. Kos M. Hagoort P. (2008). The neural integration of speaker and message. J. Cogn. Neurosci. 20, 580–591. 10.1162/jocn.2008.20054 18052777

[B44] Vangsnes Ø. A. Söderlund G. Blekesaune M. (2017). The effect of bidialectal literacy on school achievement. J. Bilingual Educ. Bilingual. 20, 346–361. 10.1080/13670050.2015.1051507

[B45] Vikør L. S. (2001). The Nordic Languages: Their Status and Interrelations. Oslo: Novus Press.

[B46] Vikør L. S. (2015). Norwegian: Bokmål vs. Nynorsk. *Språkrådet* 1–11. Available online at: http://www.sprakradet.no/Vi-og-vart/Om-oss/English-and-other-languages/English/norwegian-bokmal-vs.-nynorsk (Accessed July 27, 2018).

[B47] Volterra V. Taeschner T. (1978). The acquisition and development of language by bilingual children. J. Child Lang. 5, 311–326. 10.1017/S0305000900007492

[B48] Yan S. Kuperberg G. R. Jaeger T. F. (2017). Prediction (or not) during language processing. A commentary on Nieuwland et al. (2017) and DeLong et al. (2005). bioRxiv [preprint]. 10.1101/143750

